# Delta-9-Tetrahydrocannabinol alleviates doxorubicin-induced weight loss but does not affect dextran sodium sulphate-induced colitis

**DOI:** 10.1186/s42238-026-00386-z

**Published:** 2026-01-14

**Authors:** Malene Wiborg Johansen, Maria C. E. Andersen, Thomas Nissen, Anders B. Nexoe, Seyda Ünsal, Gunvor I. Madsen, Sören Möller, Jens Kjeldsen, Grith Lykke Sorensen, Uffe Holmskov, Steffen Husby, Mathias Rathe

**Affiliations:** 1 Hans Christian Andersen Children’s Hospital, Odense University Hospital, Kløvervænget 23C, Odense, 5000 Denmark; 2 Department of Cancer and Inflammation Research, Institute of Molecular Medicine, University of Southern Denmark, Odense, Denmark; 3 Department of Pathology, Odense University Hospital, Odense, Denmark; 4 OPEN-Open Patient Data Explorative Network, Odense University Hospital, Odense, Denmark; 5 Department of Clinical Research, University of Southern Denmark, Odense, Denmark; 6 Department of Medical Gastroenterology, Odense University Hospital, Odense, Denmark; 7 Research Unit of Medical Gastroenterology, Department of Clinical Research, University of Southern Denmark, Odense, Denmark

**Keywords:** Mucositis, Cannabinoid, Chemotherapy, Gastrointestinal toxicity, Inflammatory bowel disease, Intestinal inflammation

## Abstract

**Background:**

Gastrointestinal mucositis is a common adverse effect of chemotherapy. Inflammatory bowel diseases (IBD) are a spectrum of chronic inflammatory disorders of the digestive tract. Mucositis and IBD/colitis are characterized by weight loss and intestinal inflammation. Previous animal studies suggest that the cannabinoid, ∆9-tetrahydrocannabinol (THC), attenuates intestinal inflammation. We aimed to investigate the effect of THC oil on doxorubicin-induced gastrointestinal mucositis and dextran sodium sulphate (DSS) induced colitis in mice.

**Methods:**

Wild-type C57BL6 mice were used for the experiments. The mice received a daily dose of THC oil at either 10 mg/kg or 20 mg/kg or vehicle by oral gavage. Mucositis was induced by intraperitoneal injection of doxorubicin, while colitis was induced by supplementing the drinking water with 2% DSS. Gastrointestinal toxicity was evaluated by weight, intestinal lengths, histopathological evaluation, and expression of genes related to chemotherapy-induced mucositis and intestinal inflammation.

**Results:**

Treatment with THC significantly reduced weight loss and increased the length of the small intestine in the doxorubicin-treated mice. No effects on gene expression of pro-inflammatory cytokines were observed. No effect of THC was observed in DSS-induced colitis.

**Conclusions:**

THC reduced doxorubicin-induced weight loss, possibly due to increased appetite, but did not affect doxorubicin-induced intestinal inflammation or DSS-induced colitis in mice.

## Introduction

Mucositis is a common, debilitating adverseeffect of chemotherapeutic treatment (Sonis et al., 2004). The condition is characterized by an inflammatory reaction and damaged epithelium along the mucous membranes lining the alimentary tract causing structural functional and immunological changes (Sougiannis et al., 2021). Gastrointestinal (GI) mucositis may compromise nutritional intake nutrient absorption and digestion which can lead to malnutrition and loss of body weight (Kuiken et al., 2014). These treatment-related complications may limit the dose of chemotherapy and negatively influence the prognosis (Lalla et al., 2014). Numerous interventions have been proposed for the prevention or amelioration of mucositis. However no general treatment or preventive strategy for alimentary tract mucositis across antineoplastic regimens is currently recognized (Lalla et al., 2014; Rathe et al., 2018; Gibson et al., 2013).

Inflammatory bowel diseases (IBD) which include Crohn’s disease (CD) and ulcerative colitis (UC) represent a spectrum of chronic inflammatory disorders of the digestive tract. In IBD patients the inflammatory process in genetically susceptible individuals is believed to be sustained in part by an impaired immune response against intestinal microorganisms. The cornerstones of IBD treatment have been the therapeutic use of anti-inflammatory and immunosuppressive drugs combined with surgery (Peyrin-Biroulet et al., 2015). The treatment of IBD has been revolutionized by biological agents which are protein-based molecules that can inhibit inflammation (Tamilarasan et al., 2019; Chao and Loshak 2019) with the pro-inflammatory cytokine TNF-α as the most common target. However approximately one third of patients treated with anti-TNF-α agents experience insufficient or complete lack of response (Komaki et al., 2016; Roda et al., 2016).

In recent years there has been a surge in demand for Cannabis sativa for medical purposes. Natural or synthetic derivatives of the plant are increasingly being prescribed or self-administered for a variety of medical conditions most notably against chemotherapy-related nausea chronic pain and muscle spasms (Borgelt et al., 2013). The two main bioactive compounds found in cannabis ∆9-tetrahydrocannabinol (THC) and cannabidiol (CBD) are suggested to exert an anti-inflammatory effect although the underlying mechanisms and the interactions between these compounds and their targets is not fully elucidated (Miller et al., 2020). In a rat colitis model Jamontt et al. showed that among CBD THC and sulfasalazine THC was the most effective single agent in reducing inflammation because it significantly improved all parameters (Jamontt et al., 2010). Chemotherapy-induced mucositis and IBD share several inflammatory features, including disruption of the intestinal epithelial barrier, activation of innate immune responses, and production of pro-inflammatory cytokines.

Therefore, THC could be effective as treatment for chemotherapy-induced gastrointestinal mucositis with the associated weight loss or colitis.

In the current study we evaluated the effect of THC oil in an established model of chemotherapy-induced gastrointestinal mucositis (Andersen et al, 2020; Bech et al., 2021; Dalby et al., 2022) and used a dextran sodium sulphate (DSS) induced colitis model (Chassaing et al., 2014) to evaluate the generalizability of observations.

## Methods

The anti-inflammatory effect of THC was investigated in chemotherapy (doxorubicin/DOXO) induced mucositis and dextran sulfate sodium (DSS) induced colitis.

In the doxorubicin-study, mice were randomly assigned to one of eight groups (Table 1). The mice were treated with either saline or doxorubicin (20 mg/kg body weight) to induce mucositis by intraperitoneal (i.p.) injection and euthanized at day 2 or at day 7 post administration. The mice received a daily dose of THC oil at either 10 mg/kg body weight or 20 mg/kg body weight or vehicle by oral gavage.

**Table 1:** Overview of treatment groups and treatment group assignment in the two experiments

Groups	Included	Excluded	Included for analysis	I.p. injection	Gavage type
Doxorubicin-Study					
Control + Vehicle	5	0	5	Saline	Vehicle
Control + THC20	5	0	5	Saline	20 mg/kg THC
Doxorubicin + Vehicle (D2)	6	1	5	Doxorubicin	Vehicle
Doxorubicin + Vehicle (D7)	11	3	8	Doxorubicin	Vehicle
Doxorubicin + THC 10 mg/kg (D2)	11	1	10	Doxorubicin	10 mg/kg THC
Doxorubicin + THC 10 mg/kg (D7)	22	12	10	Doxorubicin	10 mg/kg THC
Doxorubicin + THC 20 mg/kg (D2)	13	3	10	Doxorubicin	20 mg/kg THC
Doxorubicin + THC 20 mg/kg (D7)	18	6	12	Doxorubicin	20 mg/kg THC
Total	91	26	65		
DSS-Study					
Groups	Included	Excluded	Included for analysis	Water type	Gavage type
Control + Vehicle	13	0	13	Standard	Vehicle
Control + THC20	5	0	5	Standard	20 mg/kg THC
DSS + Vehicle	5	0	5	2% DSS	Vehicle
DSS + THC10	10	0	10	2% DSS	10 mg/kg THC
DSS + THC20	17	6	11	2% DSS	20 mg/kg THC
Total	50	6	44		

Doxorubicin-study: Mice were randomly assigned to one of the eight groups. The mice were treated with either saline or doxorubicin (20 mg/kg body weight) to induce mucositis by intraperitoneal (i.p.) injection and euthanized at day 2 or at day 7 post administration. The mice received a daily dose of THC oil at either 10 mg/kg body weight or 20 mg/kg body weight or vehicle alone by oral gavage

DSS-study: Mice were randomly assigned to one of five groups. The mice were given ad libitum water with or without added dextran sulfate sodium (DSS) for the first 7 days, after which all mice were given standard water for the remaining 3 days. The mice were gavaged daily for the first 7 days with either vehicle, 10 or 20 mg/kg THC. Vehicle comprised of only corn oil

In the DSS-study, mice were randomly assigned to one of five groups (Table 1).

The mice were given ad libitum water with or without added DSS for the first 7 days, after which all mice were given standard water for the remaining 3 days. The mice were gavaged daily for the first 7 days with either vehicle or 10 or 20 mg/kg THC.

### Animals

#### Doxorubicin study

Ninety-one adult female wild-type C57BL6 mice between the ages of 10 and 12 weeks were used. Seventy-four Murine Pathogen-Free (MPF) mice were from Taconic Biosciences (Ejby, Denmark). Seventeen mice were bred in-house with mice from Taconic.

#### DSS study

Fifty male wild-type C57BL/6 mice were used. Of these, 37 were bought from Taconic Biosciences (Ejby, Denmark), while 13 were bred in-house from Taconic parents.

All animals were maintained at a 12-h light/dark cycle at constant temperature (20–24 °C) and 54–55% humidity. The mice had access to water and standard chow ad libitum. The mice were single-housed during the experiments. Cages contained woodchips bedding, nesting material, and a wood chew.

### Induction of mucositis and colitis

#### Doxorubicin study

Mucositis was induced by doxorubicin. The mice were randomized (www.random.org) into treatment and control groups receiving doxorubicin 20 mg/kg (Teva, Kongens Lyngby, Denmark) diluted 1:1 in isotonic saline administrated by a single intraperitoneal injection on day 0 or an equivalent volume of 0.9% NaCl. Euthanasia was performed at day 2 or at day 7 as described earlier (Andersen et al., 2020). Doxorubicin-treated mice experiencing weight loss < 5% were excluded from the study due to presumed injection error (*n*= 10) (Gaines Das and North 2007; Arioli and Rossi 1970). Fifteen mice were euthanized prematurely due to > 20% weight loss based on humane endpoints. One mouse was euthanized prematurely due to an abscess in the jaw (Table 1).

#### DSS study

Colitis was induced in the mice by supplementing the drinking water with 2% DSS (MP Biomedicals, Solon, OH, USA). The mice were randomly allocated to 7 days of either regular drinking water or water supplemented with 2% DSS, followed by 3 days of regular drinking water (Table 1). The dose of DSS was based on pilot studies that showed insufficient weight loss in the case of 1% and 1.5% doses of DSS. A dose of 2.5% DSS was associated with extensive weight loss (> 20%) and high mortality (data not shown).

### Administration and detection of THC

THC oil (Dronabinol, Glostrup Pharmacy, Denmark) or vehicle as corn oil (Sigma-Aldrich, St. Louis, United States) was administered by oral gavage daily. THC oil was given in two different doses, 10 mg/kg body weight and 20 mg/kg body weight. The doses were selected based on previously published work (Jamontt et al., 2010; Pagano et al., 2016) and a pilot study showing that both doses were well tolerated (data not shown). THC oil was dissolved in corn oil (100 µl/mouse) (Diehl et al., 2001). Controls received an equivalent volume of corn oil by oral gavage. In the doxorubicin study, the mice received a preloading dose of THC one day prior to the doxorubicin treatment (day −1). In total, the mice received THC for 3 or 8 consecutive days according to their groups. In the DSS-study THC was administered daily for 7 days by oral gavage. Oral gavage was performed using a 21-gauge intubation tube attached to a 1 ml syringe. The gavage procedure was performed by trained animal care staff to ensure consistency and minimize animal distress.

We analyzed urine samples for the THC’s metabolite 11-nor-∆9-tetrahydrocannabinol-9-carboxylic acid (∆9-THC-COOH). The qualitative detection was done using THC dip cards (WHPM Inc. Irwindale, California, USA) to confirm the presence of THC in mice gavaged with THC or absence of THC in mice given vehicle. This was done one day prior to euthanization in a subset of mice (*n* = 43) in the doxorubicin -study and (*n* = 14) in the DSS-study. The test yields a positive result when the concentration exceeds 50 ng/mL.

### Experimental procedures

In both experiments, body weight was measured daily.

#### Doxorubicin study

Mice were sacrificed at day 2 or at day 7 by cardiac puncture during ketamine/xylazine (100 mg/kg/10 mg/kg) anesthesia. Cardiac puncture was performed using a 1 ml syringe with a 23-gauge needle.

Perfusion was performed into the heart with 10 ml cold Bulbecco’s Phosphate Buffered Saline (BPBS) (Sigma-Aldrich, St. Louis, USA). The small intestine from the pyloric sphincter to the ileocecal junction was removed and divided into three equal segments representing the duodenum, jejunum, and ileum. Due to invagination of the small intestine, one mouse was excluded from these analyses. The colon was cut from cecum to the rectum. Subsequently, the intestinal segments were measured and washed with BPBS. The distal 1.5 cm of the segments were used for histology. The remaining parts were cut into three pieces and snap frozen in liquid nitrogen and stored at −80 °C.

#### DSS study

Water intake was measured daily for the first 7 days to ensure sufficient DSS intake. The mice were sacrificed at day 10 by CO_2_-asphyxiation and subsequent heart puncture. The intestines were dissected free and the colon-cecum length was measured. The colon was separated from the cecum and flushed with cold phosphate-buffered saline (PBS).

The most proximal 1.5 cm of the colon was fixed in formalin and used for histological scoring. The most distal part of the colon was divided into two segments, proximal and distal, and snap-frozen in liquid nitrogen prior to −80 °C storage.

### Histological preparation and evaluation

All histological samples were kept in formaldehyde 4% aqueous solution (VWR international BVBA, Leuven, Belgium) for 24 h and transferred to PBS with 0.05% NaN_3._ After fixation, the tissue was embedded in paraffin, cut into three cross-sections with a thickness of 5 µm, mounted on glass slides, and stained with Hematoxylin and Eosin (H&E) at the Department of Pathology at Odense University Hospital, Odense, Denmark.

#### Doxorubicin study

The villus height and crypt depth in the small intestine were determined in ten representative villus/crypt units per intestinal segment using scanned images from histology slides and the nano zoomer digital pathology software (ndp.view 2.4.26, Hamamatsu, Photonics, Hamamatsu City, Japan). Crypt depth was measured from the base to the crypt/villus junction and the villus height from the crypt/villus junction to the tip of the villus. A blinded observer did all measurements. All tissue scoring was performed by a blinded trained pathologist. H&E stained jejunal tissue sections were scored for mucositis as previously described (Andersen et al., 2020; Chiu et al., 1970). The mucosal damage was graded from 0 to 5.

#### DSS study

H&E stained colon tissue was scored according to a previously published system (Cooper et al., 1993) The crypt architecture (0: normal—3: severe crypt distortion with loss of entire crypts), degree of inflammatory cell infiltration (0: normal—3: dense inflammatory infiltrate), muscle thickening (0: base of crypt sits on the muscularis mucosae—3: marked muscle thickening present), goblet cell depletion (0: absent—1: present), and crypt abscess (0: absent—1: present).

### Quantitative real-time PCR (qRT-PCR) of IL-1β, IL-6, and Tnf-α

We evaluated the expression of IL-1β, IL-6, and TNF-α in the jejunum samples in the doxorubicin-study and colon samples in the DSS-study. RNA was isolated from tissue with 1 ml TRIzol Reagent (VWR International, Radnor, USA). The RNA pellet was dissolved in 50–310 μl RNase-free water and kept at − 80 °C until use. RNA was quantified, and the purity was confirmed by spectrophotometry using the NanoDrop (Thermo Scientific, Wilmington, DE, USA). All samples had A260/280 nm ratios ≥ 1.8 suggesting adequate RNA purity. Since DSS interferes with qRT-PCR analysis, the colonic RNA was further purified using lithium chloride (Sigma-Aldrich, St. Louis, MO, USA), as described previously (Kerr et al., 2012).

A total of 2 µg RNA was used for cDNA synthesis. M-MLV Reverse Transcriptase mastermix (Sigma-Aldrich, St. Louis, USA) was used in a 20 ul/reaction and incubated for 50 min at 37 °C and 10 min at 80 °C to synthesise cDNA.

Real-time qPCR was done using TaqMan Universal II, (Thermo Fisher,Waltham, USA) and TaqMan Gene Expression Assays’ IL-1β (cat. No. Mm00434228_m1), IL-6 (cat. No. Mm00446190_m1) and TNF-α (cat. No. Mm00443258_m1) (Thermo Fisher, Waltham, USA).

All samples were run in triplicates using StepOnePlus (Applied Biosystems, Foster City, CA, USA). Variations in ct-values above 0.5 were excluded. Quantification of target transcript was calculated by the 2^−∆∆Ct^ method relative to an endogenous control gene *Gapdh.* Afterwards, the qRT-PCR data were normalized to saline day 7 mice (Livak and Schmittgen 2001).

### Statistical analysis

Weight change was analyzed using two-way ANOVA followed by Bonferroni’s multiple comparison test. Mice that were euthanized prematurely due to > 20% weight loss as described in the humane endpoint were included in the weight change analysis up to the time of euthanasia. The intestinal length, were analyzed by one-way ANOVA with Bonferroni’s multiple comparison test. Quantitative real-time PCR, inflammation score and damage score data did not follow a normal distribution, as tested in the D’Agostiono-Pearson omnibus normality test. RT-PCR, inflammation score and damage score data were therefore analyzed using the nonparametric Kruskal–Wallis test.

Data are presented as means ± SEM. *P*-values < 0.05 were considered statistically significant. All statistical analyses were done using GraphPad Prism (version 7.04 for Windows, GraphPad Software, La Jolla California USA).

## Results

### THC reduces weight loss in doxorubicin-induced mucositis

**Fig. 1:**
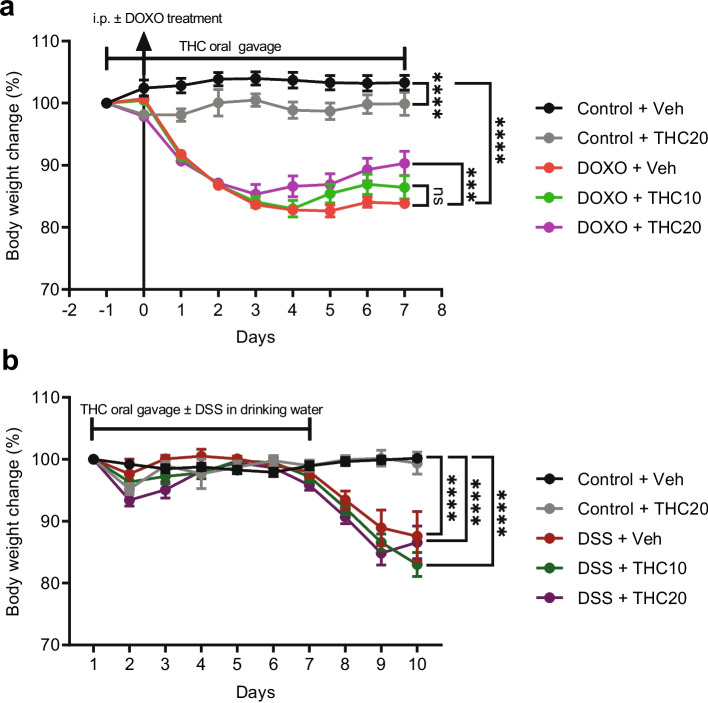
Changes in mouse body weight over time in THC-treated mucositis and colitis. **a** Weight change (%) as compared with baseline weight in Doxorubicin (DOXO)-induced mucositis.** b** Weight change (%) as compared with baseline DSS-induced colitis. Veh = vehicle treatment, THC10 = 10 mg/kg THC, THC20 = 20 mg/kg THC. Results are presented as means ± SEM. Weight is relative to baseline weight on day −1. ***p ≤ 0.001, ****p ≤ 0.0001

Significant weight loss was observed in the groups treated with doxorubicin (*p* = 0.0001; Fig. 1a). Mice in the doxorubicin-treated + 20 mg/kg THC group lost significantly less weight than the doxorubicin + vehicle group from day 1 until day 7 (*p* = 0.0002; Fig. 1a). Treatment with 20 mg/kg THC in the control group significantly reduced body weight compared to the control + vehicle mice (*p* = 0.0001; Fig. 1a). No body weight difference was observed between the doxorubicin + 10 mg/kg THC and doxorubicin + vehicle group.

### THC does not affect weight loss in DSS-induced mucositis

Significant weight loss was observed in the groups treated with DSS (*p* = 0.0001; Fig. 1b). However, treatment with THC did not significantly affect the body weight change (Fig. 1b). THC did not affect the weight in the control group in the DSS-experiment.

### THC increases small intestinal length in doxorubicin-induced mucositis

**Fig. 2:**
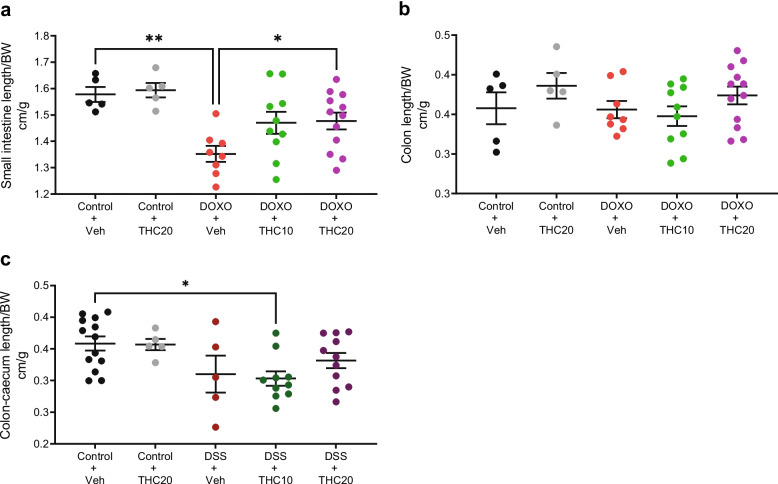
Changes in mouse intestinal length in THC-treated mucositis and colitis. **a** Small intestinal length on day 7 after start of DOXO-treatment. **b** Length of the colon on day 7 after start of DOXO-treatment. **c** Colon-caecum intestinal length at day 10 in the DSS study. BW = Body weight, Veh = vehicle treatment, THC10 = 10 mg/kg THC, THC20 = 20 mg/kg THC. Results are expressed as means ± SEM. The intestinal lengths are shown relative to baseline weight. *p < 0.05, **p ≤ 0.01

Small intestinal length was not significantly reduced by doxorubicin treatment at day 2 (data not shown). However, it was significantly shortened by doxorubicin treatment compared to saline at day 7 (*p* = 0.002; Fig. 2a). 20 mg/kg THC significantly increased the length of the small intestine at day 7 compared to the doxorubicin + vehicle (*p* = 0.048; Fig. 2a). There was no significant difference in the colon length with either treatment (Fig. 2b).

### THC does not affect the colon length in DSS-induced mucositis

The colon-caecum length was significantly decreased in the DSS + 10 mg/kg THC group compared to vehicle control (*p* = 0.027; Fig. 2c). The length of the colon was not significantly changed in the DSS + THC treated mouse groups compared to the DSS + vehicle group (Fig. 2c).

### THC treatment did not affect the mucositis score or the histological damage score, in doxorubicin- and DSS-induced mucositis, respectively

**Fig. 3:**
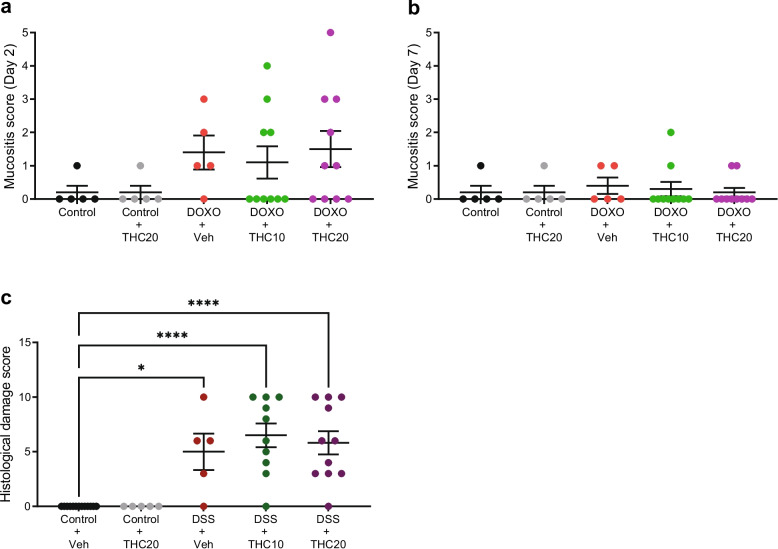
Mucositis and histological damage scores. **a** Mucositis score at day 2 in the doxorubicin (DOXO)-experiment. **b** Mucositis score at day 7 in the DOXO-experiment. **c** Histological damage score in the DSS-experiment. Veh = vehicle treatment, THC10 = 10 mg/kg THC, THC20 = 20 mg/kg THC. Results are presented as means ± SEM. *p < 0.05, ***p ≤ 0.001, ****p ≤ 0.0001

No significant differences between treatment groups were seen in the mucositis score following doxorubicin treatment at day 2 or day 7, even if a tendentially higher score was observed at day 2 (Fig. 3a and b).

A significant increase in the histological damage score was observed in groups treated with DSS, an effect which was independent of THC-treatment (*p* = 0.012 for control + vehicle versus DSS + vehicle, *p* = 0.0001 for both control + vehicle versus DSS + THC10 and DSS + THC20, Fig. 3c

### THC treatment did not decrease expression of mRNA levels of IL-1β, IL-6, or TNF-α after doxorubicin-treatment

**Fig. 4:**
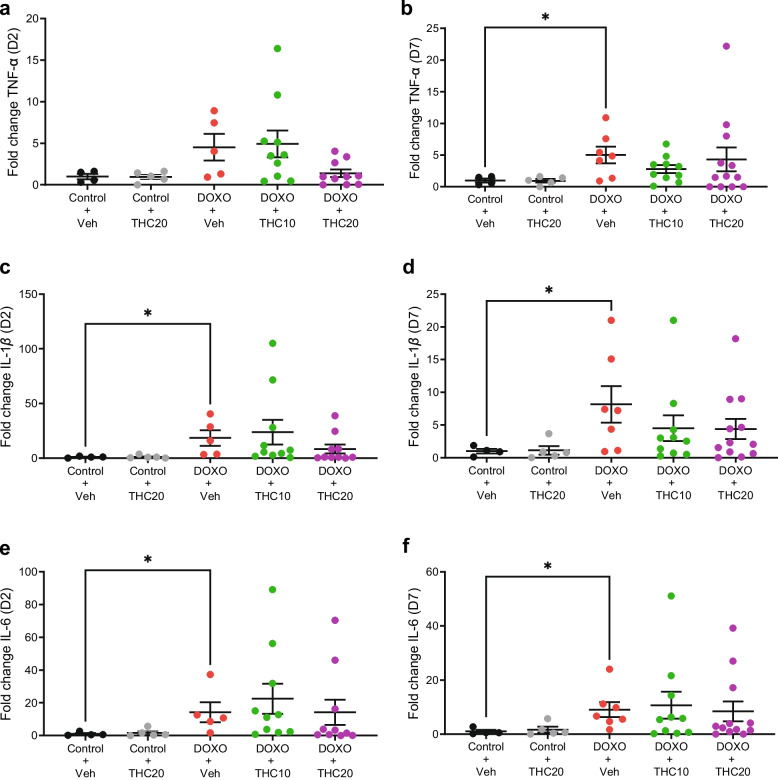
Effects of THC on expression of pro-inflammatory cytokines determined by qRT-PCR in doxorubicin-treated animals. (**a, b**) jejunal expression of TNF-alpha, (**c, d**) jejunal expression of IL-1 beta, (**e, f**) jejunal expression of IL-6 at day 2 and day 7 respectively. Note the difference in the y-axis scale among the cytokines. Veh = vehicle treatment, THC10 = 10 mg/kg THC, THC20 = 20 mg/kg THC. Data are shown as relative gene expression in the mean fold change ± SEM. *p < 0.05

The mRNA expression of IL-1β, IL-6, and TNF-α was determined in jejunal tissue at day 2 and at day 7 after start of doxorubicin treatment. No significant overexpression of TNF-α was seen in doxorubicin-treated mice on day 2 (Fig. 4a), while TNF-α was significantly increased with doxorubicin treatment compared to saline at day 7 (*p* = 0.044; Fig. 4b). Doxorubicin treatment resulted in a significant increase in IL-1β expression at day 2 (*p* = 0.031; Fig. 4c) and day 7 (*p* = 0.029; Fig. 4d). Likewise, doxorubicin treatment resulted in a significant increase in IL-6 expression at day 2 (*p* = 0.044; Fig. 4e) and day 7 (*p* = 0.017; Fig. 4f). THC treatment did not significantly alter the gene expression of IL-1β, IL-6, or TNF-α compared to vehicle treatment (Fig. 4a-f).

### THC treatment did not decrease expression of mRNA levels of IL-1β, IL-6, or TNF-α after DSS-treatment

**Fig. 5:**
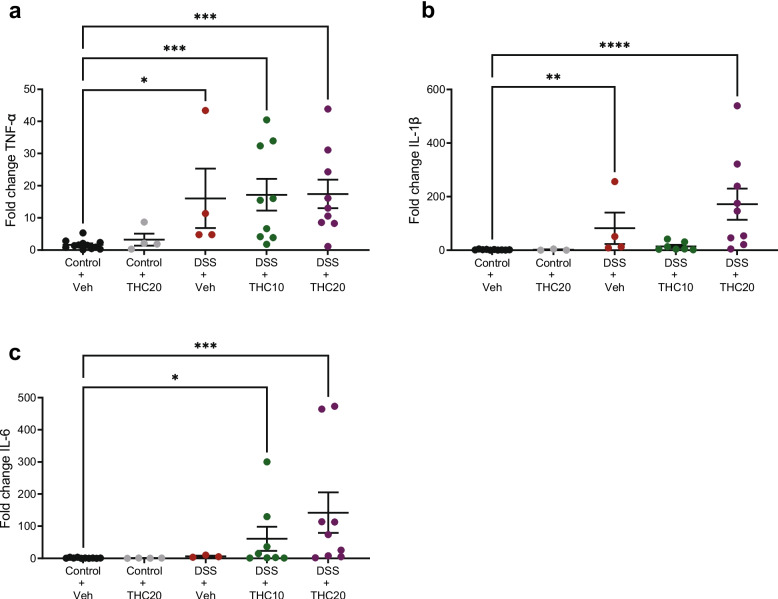
Effects of THC on expression of pro-inflammatory cytokines determined by qRT-PCR in DSS-treated animals. **a-c** represents the data from IL-1 beta, TNF-alpha and IL-6 respectively. Note the difference in the y-axis scale among the cytokines. Veh = vehicle treatment, THC10 = 10 mg/kg THC, THC20 = 20 mg/kg THC. Data are shown as relative gene expression in the mean fold change ± SEM. *p < 0.05, **p ≤ 0.01, ***p ≤ 0.001, ****p ≤ 0.0001

In all DSS-treated groups, a significant overexpression of TNF-α was observed (*p* = 0.01 for vehicle, *p* = 0.0007 for THC10, and *p* = 0.0003 for THC20; Fig. 5a). Treatment with THC did not significantly affect TNF-α expression. The level of IL-1β was significantly elevated in the DSS + vehicle and DSS + 20 mg/kg THC group (*p* = 0.006 for vehicle and *p* = 0.0001 for THC20; Fig. 5b), but treatment with THC did not affect this. Furthermore, the level of IL-6 was elevated in the DSS + 10 mg/kg THC and DSS + THC20 groups (*p* = 0.017 for THC10 and *p* = 0.0002 for THC20 Fig. 5c). THC did not alter the IL-6 expression.

### Detection of THC metabolite in the mouse urine

The main THC metabolite excreted in the urine is ∆9-THC-COOH (Hayley et al., 2018).

In the doxorubicin experiment 100% of the animals tested in the 20 mg/kg THC group tested positive for the presence of the metabolite one day prior to euthanization (*n* = 18). In the 10 mg/kg THC group, 59% tested positive (*n* = 17). All control animals tested were negative (*n* = 8).

In the DSS-experiment, the urine of a total of 14 mice was tested for the presence of THC. Of the six that tested positive, all were true positives. Of the eight that tested negative, four were true negatives, whereas four had received THC.

## Discussion

In this study, we investigated the effect of THC administered in two different doses on doxorubicin-induced mucositis and DSS-induced colitis in mice.

THC had no significant effect on inflammatory cytokine expression or histological mucositis scores in doxorubicin treated animals. However, we did find an effect of THC in the dose of 20 mg/kg on the reduction of doxorubicin-induced weight loss. Furthermore, doxorubicin treated animals that received THC had significantly longer small intestines at day 7 compared to doxorubicin-treated mice receiving vehicle. These effects were apparently model-specific as no similar effects on weight loss or intestinal length were observed in the DSS model within a similar timeframe. The difference may relate to DSS-induced colitis being more severe (markedly higher histological damage) compared with the gastrointestinal inflammation seen in the doxorubicin model.

Mice treated with doxorubicin showed weight loss at days 0–3 followed by a period of stagnating weight. The weight curve development and the observed reduction of small intestine length are in accordance with similar studies from our group (Andersen et al., 2020; Bech et al., 2021). The doxorubicin treatment affected the small intestine more than the colon. Accordingly, the mucosal morphometry of the duodenal segment was more affected by the doxorubicin than the more distal segments of the small intestine. These observations are also in accordance with previous studies, data not shown (Andersen et al., 2020).

Expression of NF-KB mediated genes and production of pro-inflammatory cytokines such as IL-1 β, IL-6, and TNF-α are involved in developing chemotherapy induced mucositis (Sonis 2004). In line with previous studies, we demonstrated an association between elevated IL-1β, IL-6, and TNF-α levels and treatment with a chemotherapeutic agent (Logan et al., 2008; Yeung et al., 2015). All three genes are also related to chemotherapy induced mucositis and inflammation (Logan et al., 2008; 2009). In addition, a previous study found associations between DSS and elevated pro-inflammation cytokines such as IL-1β, IL-6, and TNF-α (Kim et al., 2012). Doxorubicin- and DSS-induced inflammatory cytokine expression was not affected by THC treatment.

There is evidence to suggest that cannabinoids, including THC, improve chemotherapy-induced nausea and vomiting (Allan et al., 2018; Badowski 2017). Furthermore, studies have reported an analgesic effect of THC, but a more recent review suggests a limited pain-relieving effect of THC (Stockings et al., 2018). THC is well known as an appetite stimulator, and it is used therapeutically to treat anorexia and cachexia in cancer patients (Badowski and Yanful 2018). According to previous studies, THC increased food intake at doses up to 10 mg/kg and decreased food intake in higher doses of 30 mg/kg (Koch 2001; Wiley et al., 2005). We observed a weight loss in the 20 mg/kg THC treated control group suggesting some variation in the food intake-regulatory effects of commercially available THC. The body weight regulatory effect was only observed in the doxorubicin-induced model of chemotherapy-induced mucositis and not in the DSS model, suggesting the effect is not consistent at such doses or across different clinical scenarios. A recent review highlights that the relationship between cannabis and weight is complex and although THC is often linked to increased appetite and sedentary behavior, most studies show cannabis use is not associated with weight gain (Goodpaster 2025).

Despite the effect of THC on the intestinal length, our combined data suggest that THC did not alleviate the gastrointestinal mucositis due to no effect in histological changes or on the gene expression of pro-inflammatory cytokines.

To our knowledge, the effects of cannabinoids in a mucositis animal-model have only been investigated in few previous studies (Abalo et al., 2017; Yang et al., 2024). In parallel with our study, the study found that the cannabinoid agonist WIN 55,212–2 did not prevent intestinal mucositis after administration of the chemotherapeutic agent 5-fluoruracil. However, the study did suggest an effect on chemotherapy-induced diarrhea where as a recent study suggest that CBD ameliorates paclitaxel induced oral mucositis in mice (Yang et al., 2024).

The current study has some limitations. We did not monitor the food intake of the animals, limiting interpretation of weight changes. Additionally, the study was not designed to elucidate mechanisms underlying THC's effects, which should be addressed in future research. However, our main objective was to evaluate the effect on GI inflammation and THC had no effect on this in either experiment. Furthermore, intraperitoneal administration of doxorubicin poses a risk of inadvertent administration in hollow organs. Accordingly, mice experiencing a weight loss of < 5% were excluded from the study. Error rates corresponded to what was previously reported (Gaines Das and North 2007; Arioli and Rossi 1970). Detection of THC in the urine of the THC treated mice is considered a strength in the study. Most of the mice had a detectable level of excreted ∆9-THC-COOH, and we consider this proof of concept regarding the oral administration of THC (Schlienz et al., 2018). We acknowledge that LC–MS/MS or GC–MS would provide a more precise quantification. However, dip cards were used as a qualitative confirmation of oral THC intake, not for pharmacokinetic purposes. The occasional negatives in the THC10 group likely reflect dose-related variability and assay detection limits rather than absence of exposure.

In conclusion, in doxorubicin treated mice oral administration of the cannabinoid THC reduced weight loss in the 20 mg/kg THC group. THC treatment resulted in an increase of small intestinal length compared to vehicle but did not affect the expression of pro-inflammatory cytokines. The data suggested that THC treatment does not ameliorate mucositis but may be beneficial for overall maintenance of body weight after doxorubicin intervention. However, these data are not unequivocal, as THC led to significant weight loss in control animals. In the DSS-study, no beneficial effect on weight loss, histological damage score, colon length, or levels of pro-inflammatory cytokines was observed. Thus, no ameliorating effect of orally administered THC on DSS-induced colitis was observed in this study.

## Data Availability

Data are available through the corresponding author.

## Abbreviations

BPBS: Bulbecco’s Phosphate Buffered Saline
CD: Crohn’s disease
DOXO: Doxorubicin
DSS: Dextran sodium sulphate
H&E: Hematoxylin and Eosin
IBD: Inflammatory bowel diseases
i.p.: Intraperitoneal
MPF: Murine Pathogen-Free
PCR: Polymerase chain reaction
THC: Tetrahydrocannabinol
UC: Ulcerative Colitis
